# Association of chemotherapy-induced nausea and vomiting or anorexia with plasma levels of five gastrointestinal peptides in patients receiving chemotherapy

**DOI:** 10.1186/s40780-025-00424-7

**Published:** 2025-03-05

**Authors:** Ryosuke Tatsuta, Ryota Tanaka, Asami Tashibu, Yosuke Suzuki, Kosuke Suzuki, Tomotaka Shibata, Tadasuke Ando, Toshitaka Shin, Yuhki Sato, Hiroki Itoh

**Affiliations:** 1 Department of Clinical Pharmacy, Oita University Hospital, Yufu, Oita Japan; 2 Department of Gastroenterological and Pediatric Surgery, Oita University Faculty of Medicine, Yufu, Oita Japan; 3 Department of Urology, Oita University Faculty of Medicine, Yufu, Oita Japan

**Keywords:** Chemotherapy-induced nausea and vomiting, Anorexia, Gastrointestinal peptides, Motilin, Leptin

## Abstract

**Background:**

Imbalance between gastrointestinal peptides has been implicated as a cause of chemotherapy-induced nausea and vomiting (CINV) and anorexia in cancer patients. This study comprehensively evaluated the changes in blood levels of five gastrointestinal peptide: substance P, neuropeptide (NPY), motilin, ghrelin and leptin, following chemotherapy, and the relationship between these peptides and CINV or anorexia.

**Methods:**

This single-center, prospective, observational study recruited 20 patients with esophageal cancer, urothelial cancer, or testiculoma undergoing cisplatin-based chemotherapy. Plasma levels of five gastrointestinal peptides were measured on days 1 (baseline; before administering chemotherapy), 3, 5 and 8 of the chemotherapy session. Anorexia and CINV were defined as visual analog scale scores 25 mm or higher at least once during the observation period.

**Results:**

Plasma NPY and leptin were significantly elevated in the early phase (day 3) of the chemotherapy session, while plasma motilin and substance P were significantly elevated in the late phase (days 5 and 8). Plasma motilin showed significant elevation on days 5 and 8 compared to baseline in CINV group but no significant increase in non-CINV group, and the levels were significantly higher in CINV than in non-CINV group. Plasma leptin peaked significantly on day 3 in both anorexia and non-anorexia groups, and remained significantly higher on day 5 compared to baseline in anorexia group but not in non-anorexia group.

**Conclusion:**

CINV is associated with excessive secretion of motilin and anorexia is related to sustained elevation of leptin, suggesting the potential of these peptides as quantitative indicators of CINV and anorexia.

**Supplementary Information:**

The online version contains supplementary material available at 10.1186/s40780-025-00424-7.

## Background

Gastrointestinal dysfunction, especially chemotherapy-induced nausea and vomiting (CINV), is an undesirable adverse effect of chemotherapy. Since anorexia caused by CINV worsens patients’ nutritional status and significantly lowers quality of life, management of CINV is important [1, 2]. Currently, the common treatment for CINV is the combined use of three agents; neurokinin 1 (NK1) receptor antagonist with serotonin receptor antagonist and corticosteroids, but the clinical efficacy of this therapy remains inadequate [3, 4]. Hence, the development of new drugs and evidence-based therapies is desired. The NCI Common Terminology Criteria for Adverse Events (CTCAE) and questionnaires such as visual analog scale (VAS) are commonly used to evaluate the severity of CINV. Disadvantages of these methods are the inability to quantify gastrointestinal dysfunction in children and patients with decreased level of consciousness, sensitivity to individual differences and the environment, and preference for numbers [5]. Therefore, it is necessary to fully elucidate the mechanisms of CINV and anorexia and to search for biomarkers that serve as quantitative indicators.

Physiological functions such as gastrointestinal motility and appetite are controlled by the coordination of the neuro-endocrine-immune system. In recent years, the pathological mechanisms of several gastrointestinal disorders have been elucidated from the viewpoint of gastrointestinal peptides such as ghrelin, leptin, substance P, motilin, and neuropeptide Y (NPY). Ghrelin is a peptide composed of 28 amino acid residues and exists in two forms; acyl-ghrelin and desacyl-ghrelin, which differ in the side chains [6]. Ghrelin is mainly produced by gastric endocrine cells and has important effects on energy metabolism control such as promotion of food intake, weight gain, and gastrointestinal tract function control [7]. In contrast, leptin strongly suppresses oral food intake and enhances energy expenditure mainly via hypothalamic receptors, and has various physiological functions such as blood pressure control and immune regulation [8]. Substance P, an 11-residue peptide, is present in primary sensory nerves, central nerves and digestive organs, and has various physiological functions such as promoting intestinal motility and bronchoconstriction [9]. Substance P is known to induce CINV through the NK1 receptor on the vomiting center nerve [10]. Therefore, aprepitant, a highly selective antagonist of NK1 receptors, is clinically recommended to be used in combination with 5-hydroxytryptamine 3 (5-HT3) receptor antagonist and dexamethasone to prevent nausea/vomiting induced by highly and moderately emetogenic cancer chemotherapy [10, 11]. Motilin accelerates gastric emptying, especially during the interdigestive state [12]. This peptide is one of the most important factors controlling the regular occurrence of phase 3 contractions of the migrating motor complex (MMC) [13]. The brain, specifically the hypothalamus, is the organ responsible for maintaining the balance between food intake and energy expenditure by receiving peripheral signals from the gastrointestinal tract. Several neuropeptides are involved in this process, such as neuropeptide Y (NPY), which increases food consumption and decreases energy expenditure [14]. Acting through binding with specific receptors, NPY plays an important role in several physiological functions including cardiovascular homeostasis, regulation of the sympathetic nervous system activity and appetite regulation [15, 16]. However, it remains unclear whether there are changes in the kinetics of plasma levels of gastrointestinal peptides after administration of chemotherapeutic agents.

Given this background, this study aimed to comprehensively evaluate the changes in blood levels of various gastrointestinal peptides (substance P, NPY, motilin, ghrelin, and leptin) after anticancer drug administration, as well as the relationship between changes in these levels and CINV or chemotherapy-induced anorexia.

## Methods

### Patient eligibility

This single-center, prospective, observational clinical study enrolled 10 patients with esophageal cancer treated with cisplatin-based chemotherapy at Oita University Hospital between April 2017 and April 2018, and 10 patients with urothelial cancer or testiculoma undergoing cisplatin-based chemotherapy between May 2019 and April 2021. The inclusion criteria were hospitalized patients; aged above 20 years; Eastern Cooperative Oncology Group performance status 0–1; preserved organ function sufficient to allow chemotherapy; written informed consent available; and treated with cisplatin. Cisplatin is an anticancer drug with a high risk of emesis, and the 2015 Japan Society of Clinical Oncology Clinical Practice clinical practice guidelines for antiemetics recommend the concomitant use of NK1 receptor antagonist, 5-HT3 antagonist and dexamethasone as antiemetics for cisplatin [17]. Patients with nausea and vomiting in the 24-h period prior to chemotherapy were excluded.

### Chemotherapeutic regimens

Patients with esophageal cancer were administered cisplatin 70–80 mg/m^2^ as a 2-h intravenous infusion on day 1 and 5-fluorouracil 700–800 mg/m^2^ as a 120-h continuous intravenous infusion from day 1 to day 5 (CDDP/5-FU). Supportive therapy and prophylaxis against expected adverse effects were provided. On day 1, intravenous infusions of fosaprepitant (NK1 receptor antagonist) 150 mg, palonosetron (5-HT3 antagonist) 0.75 mg, and dexamethasone 9.9 mg were given before cisplatin. On days 2–4, dexamethasone 8.25 mg was administered intravenously. Patients with urothelial cancer received cisplatin 70 mg/m^2^ as a 3.5-h intravenous infusion on day 2, and gemcitabine 1000 mg/m^2^ as a 30-min intravenous infusion on days 1, 8 and 15 (CDDP + GEM). On day 1, an intravenous infusion of dexamethasone 6.6 mg was given before gemcitabine. On day 2, intravenous infusions of fosaprepitant 150 mg, palonosetron 0.75 mg, and dexamethasone 9.9 mg were given before cisplatin. On days 3–5, dexamethasone 8.25 mg was administered intravenously. Patients with testiculoma received cisplatin 20 mg/m^2^ as a 3-h intravenous infusion from day 1 to day 5, etoposide 100 mg/m^2^ as a 2-h intravenous infusion from day 1 to day 5, and bleomycin 30 mg/body as a 1-h intravenous infusion on days 2, 9 and 16 (CDDP + BLM + ETP). On day 1, intravenous infusions of fosaprepitant 150 mg, palonosetron 0.75 mg, and dexamethasone 9.9 mg were given before cisplatin. On days 2–5, dexamethasone 8.25 mg was administered intravenously. No patients were using concomitant medications that affect antiemetic therapy.

### Assessment and definition of CINV and anorexia

The antiemetic effect, food intake, and use of other antiemetic agents were monitored from day 1 to day 8 of the chemotherapy session. CINV symptoms were assessed from day 1 after cisplatin administration through day 8 based on patient responses to the following questions in a diary: "Did you experience nausea within 24 h of treatment?" and "Did you vomit within 24 h of treatment?”. A VAS was used to score the severity of CINV every day, and each patient marked on a 100-mm horizontal line from 0 (none) to 100 (maximum). CINV was defined as a VAS score of 25 mm or longer at least once during the observation period, according to previous reports [18, 19]. An appetite VAS was scored from 0 (good appetite) to 100 (no appetite) three times daily after each meal, and each patient rated appetite subjectively. Daily appetite VAS score was calculated as the average of the three meals. Anorexia was defined as a VAS score of 25 mm or higher at least once during the observation period, similar to the CINV definition.

### Blood sampling and quantification of plasma gastrointestinal peptide levels

Blood sampling was performed before breakfast after an overnight fast on days 1 (before chemotherapy administration), 3, 5 and 8 of the chemotherapy session. Blood samples were collected into chilled tubes containing aprotinin (500 kallikrein inhibitor units/ml) and ethylenediaminetetraacetic acid (1.2 mg/ml). The samples were centrifuged at approximately 3000 rpm for 10 min at 4ºC. Then, the plasma was transferred to a polypropylene tube and stored frozen at −40ºC. Plasma samples for ghrelin measurement were mixed with 10% volume of 1 N hydrochloric acid. Plasma gastrointestinal levels of gastrointestinal peptides (acyl-ghrelin, leptin, NPY, motilin, and substance P) were measured by the following ELISA kits according to protocols of manufacturers: Acyl-ghrelin (human) Express ELISA kit (Bertin Pharma Inc., Montigny-le-Bretonneux, France), Human Leptin ELISA Kit (Abcam, Cambridge, UK), Human Neuropeptide Y ELISA Kit (Cloud-Clone Corp., Houston, USA), Human Motilin ELISA Kit (LifeSpan BioSciences, Inc., Seattle, USA) and Human Substance P ELISA Kit (Peninsula Laboratories International, Inc., San Carlos, USA). Plasma levels of all five gastrointestinal peptides were expressed in pg/mL. Fluorescence intensity was measured using a microplate reader (SH-9000, CORONA ELECTRIC Co., Ltd, Hitachinaka, Japan).

### Data and statistical analyses

For patient demographic and laboratory parameters, categorical data are expressed as number (%) and continuous data as median [interquartile range]. The fold changes in plasma gastrointestinal peptide levels on days 3, 5 or 8 with respect to baseline (day 1) were analyzed using Dunnett's test. Differences between the CINV and non-CINV groups as well as between the anorexia and non-anorexia groups were analyzed by chi-squared or Fisher's exact test for categorical variables and Kruskal–Wallis test with Bonferroni correction for continuous variables, excluding patients with nausea and anorexia at baseline (VAS score on day 1 ≥ 25 mm). The effect sizes of all the statistical tests were calculated using Cohen’s d and summarized in Table S1. *P* values less than 0.05 were considered statistically significant. Statistical analyses were performed using the SPSS software package (version 27.0; SPSS Inc., IL, US).

### Ethics

This study was performed in accordance with the Declaration of Helsinki and its amendments. The protocol was approved by the Ethical Review Board of Oita University Faculty of Medicine (approval numbers: 1196 and 1348). Each patient received information about the scientific aim and procedures of this study and provided written informed consent.

## Results

### Patient characteristics and VAS scores for nausea and appetite following chemotherapy

**Fig. 1:**
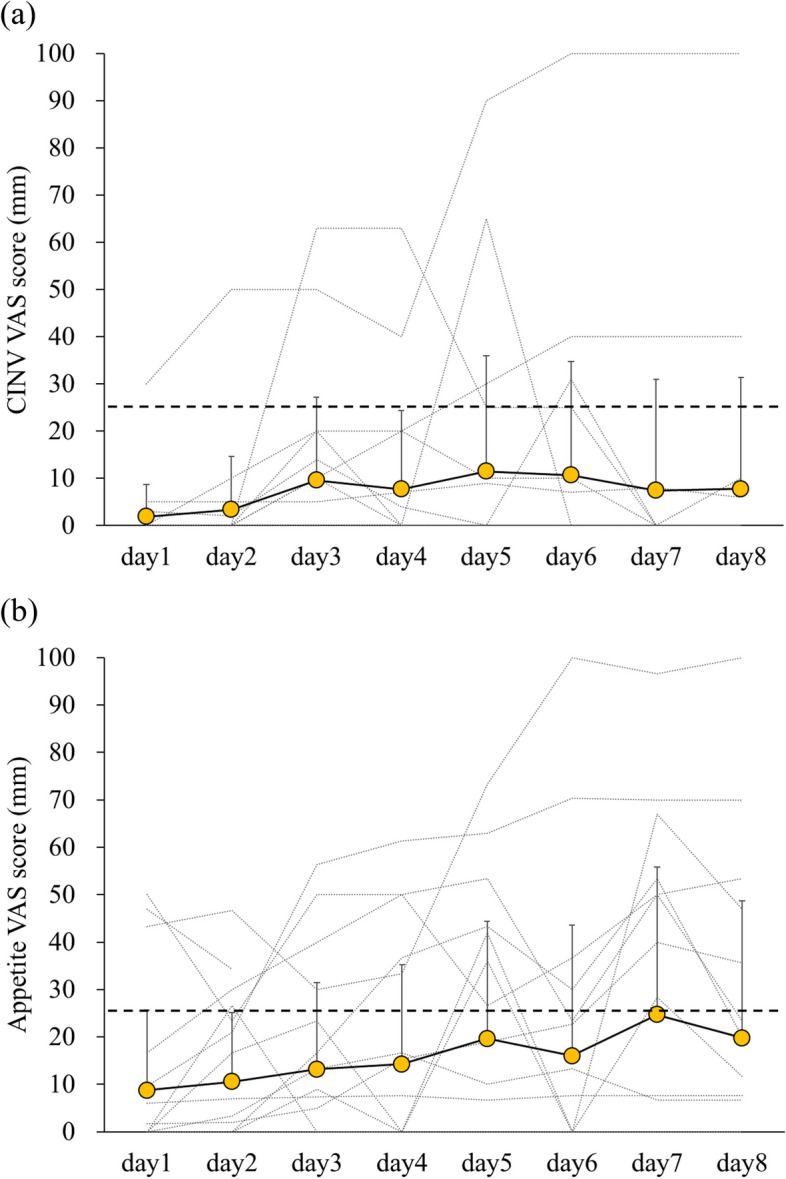
Changes in visual assessment scale scores for (**a**) chemotherapy-induced nausea and vomiting and (**b**) appetite during the observation period in all the patients studied (n = 20). Data are expressed as mean + standard deviation. Dotted lines represent the changes in individual patients. The dashed horizontal line represents 25 mm, threshold for definition of CINV or anorexia. CINV, chemotherapy-induced nausea and vomiting; VAS, visual assessment scale

Table 1 summarizes the demographic and laboratory parameters of the 20 patients studied. Sixteen males and 4 females with a median age of 70.0 years were enrolled in the study. The most frequent regimens were cisplatin plus 5-fluorouracil plus radiotherapy, and cisplatin plus gemcitabine. Of the 20 patients enrolled, one patient had nausea and three patients had anorexia at baseline (day 1) prior to chemotherapy administration (Fig. 1) and were excluded from the analysis of CINV and anorexia. Four and seven patients met the definitions of CINV and anorexia, respectively, but there were no significant increases in CINV VAS scores and appetite VAS scores compared to baseline throughout the observation period. There were no significant differences in patient background and clinical laboratory data between the CINV (*n* = 4) and non-CINV (*n* = 15) groups, and between the anorexia (*n* = 7) and non-anorexia (*n* = 10) groups (Table 1).

### Fold changes in plasma gastrointestinal peptide levels following chemotherapy

**Table 1:** Patient characteristics and clinical laboratory data

Characteristics	All	CINV		P*	Anorexia		P*
	*n* = 20	( +) *n* = 4	(-) *n* = 15		( +) *n* = 7	(-) *n* = 10	
Age (year)	70.0 [68.8–72.8]	70.0 [69.8–71.3]	70.0 [68.5–73.5]	1.00	70.0 [69.0–76.0]	70.0 [68.5–71.0]	0.67
Sex (male/female)				0.35			0.44
Male	16 (80.0)	4 (100.0)	11 (73.3)		6 (85.7)	7 (70.0)	
Female	4 (20.0)	0 (0.0)	4 (26.7)		1 (14.3)	3 (30.0)	
Body weight (kg)	60.2 [53.7–65.2]	62.0 [57.0–63.8]	59.2 [53.3–64.4]	0.89	54.8 [50.9–61.2]	60.9 [54.6–64.8]	0.36
Serum albumin (g/dL)	3.7 [3.4–3.9]	3.7 [3.4–3.9]	3.7 [3.4–3.9]	0.89	3.4 [3.3–3.7]	3.9[3.6–4.0]	0.06
BUN (mg/dL)	12.6 [11.7–15.8]	12.2 [11.6–13.9]	13.4 [11.8–16.1]	0.81	12.5 [11.8–15.1]	13.0 [11.8–15.5]	0.89
Regimen				0.48			0.58
CDDP/5-FU	2 (10.0)	0 (0.0)	2 (13.3)		1 (14.3)	1 (10.0)	
CDDP/5-FU + Radiation	8 (40.0)	3 (75.0)	5 (33.3)		4 (57.1)	3 (30.0)	
CDDP + GEM	8 (40.0)	1 (25.0)	7 (46.7)		2 (28.6)	5 (50.0)	
CDDP + BLM + ETP	2 (10.0)	0 (0.0)	1 (6.7)		0 (0.0)	1 (10.0)	
Chemotherapy course				0.72			0.63
First chemotherapy course	8 (40.0)	2 (50.0)	6 (40.0)		2 (28.6)	4 (40.0)	
Subsequent chemotherapy course	12 (60.0)	2 (50.0)	9 (60.0)		5 (71.4)	6 (60.0)	

Data are expressed as numbers (%) for categorical variables and median [interquartile range] for continuous variables. Statistical differences between the groups were analyzed using chi-squared or Fisher's exact test for categorical variables and Mann–Whitney *U* test for continuous variables. *CINV* Chemotherapy-induced nausea and vomiting, *BUN* Blood urea nitrogen, *CDDP* Cisplatin, *5-FU* 5-fluorouracil, *GEM* Gemcitabine, *BLM* Bleomycin, *ETP* Etoposide

**Fig. 2:**
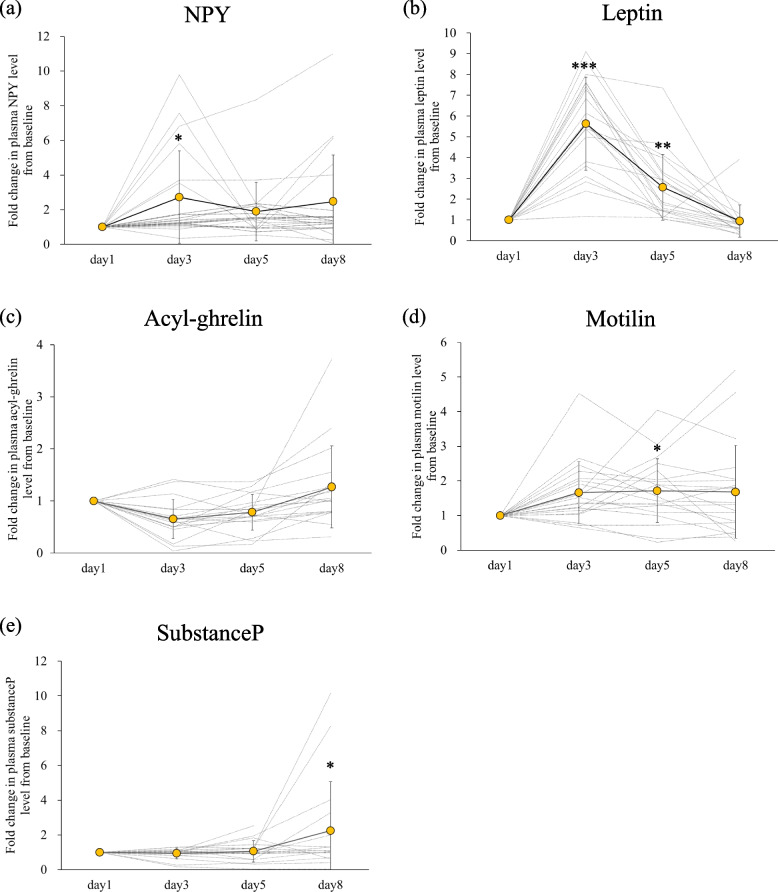
Fold changes in plasma (**a**) neuropeptide Y, (**b**) leptin, (**c**) acyl-ghrelin, (**d**) motilin, and (**e**) substance P levels with respect to baseline (day 1, before administration of chemotherapy). Dotted lines represent changes in individual patients (n = 20). Group data are expressed as mean (yellow circle) ± standard deviation (vertical bar). Significant differences between groups were analyzed by Dunnett’s test. *p < 0.05, **p < 0.01, ***p < 0.001, compared to day 1. NPY, neuropeptide Y

Figure 2 shows the fold changes in plasma levels of five gastrointestinal peptides on days 3, 5 and 8 with respect to baseline (day 1). The measured baseline levels of acyl-ghrelin in 2 patients and substance P in 2 patients were below the lower limits of quantitation of the respective ELISA kits. Plasma NPY increased significantly on day 3, and plasma leptin increased significantly on days 3 and 5 compared to baseline (Fig. 2a and b). Plasma acyl-ghrelin decreased in 15 of 18 patients on day 3, showing a trend of decrease compared to baseline (*p* = 0.065) (Fig. 2c). Plasma motilin increased significantly on day 5 and substance P level increased significantly on day 8 (Fig. 2d and e). Indicators of nutritional status did not change significantly following chemotherapy (data not shown). Although oral food intake decreased during chemotherapy, serum albumin did not decrease (3.5 ± 0.4 g/dL on day 1 and 3.4 ± 0.4 g/dL on day 8).

### Differences in plasma gastrointestinal peptide levels following chemotherapy between CINV and non-CINV

**Fig. 3:**
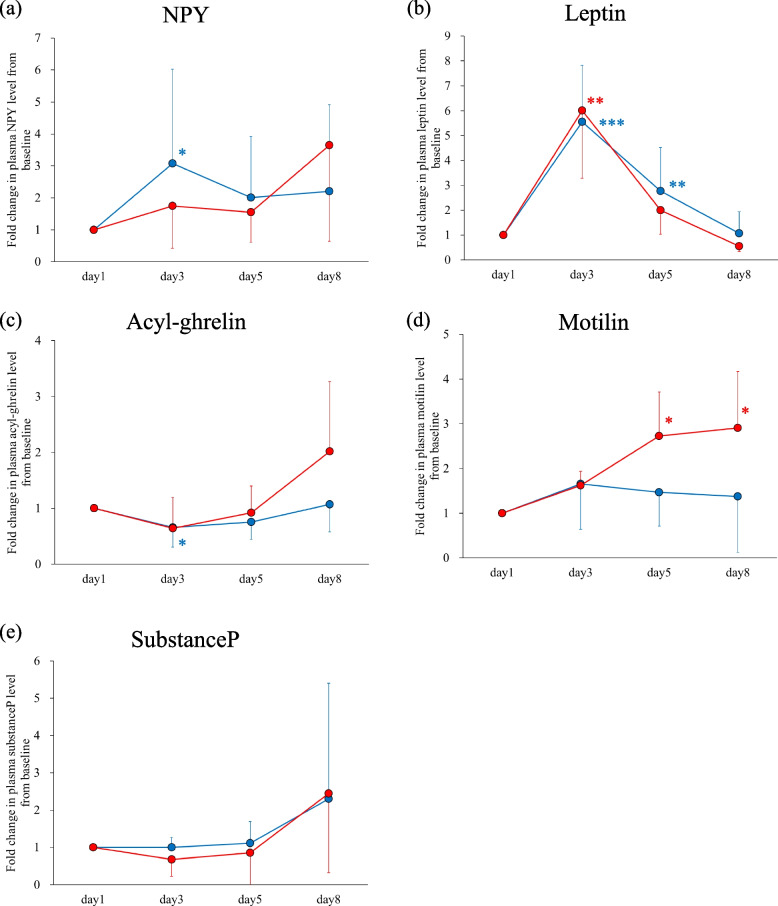
Fold changes in plasma (**a**) NPY, (**b**) leptin, (**c**) acyl-ghrelin, (**d**) motilin, and (**e**) substance P levels with respect to baseline (day 1, before administration of chemotherapy) in the CINV (red circles and lines; n = 4) and non-CINV (blue circles and lines; *n* = 15) groups. Data are expressed as mean (circle) ± standard deviation (vertical bar). Significant differences between groups were analyzed by Dunnett's test. *p < 0.05, **p < 0.01, ***p < 0.001, compared with day 1. NPY, neuropeptide Y; CINV, chemotherapy-induced nausea and vomiting

**Fig. 4:**
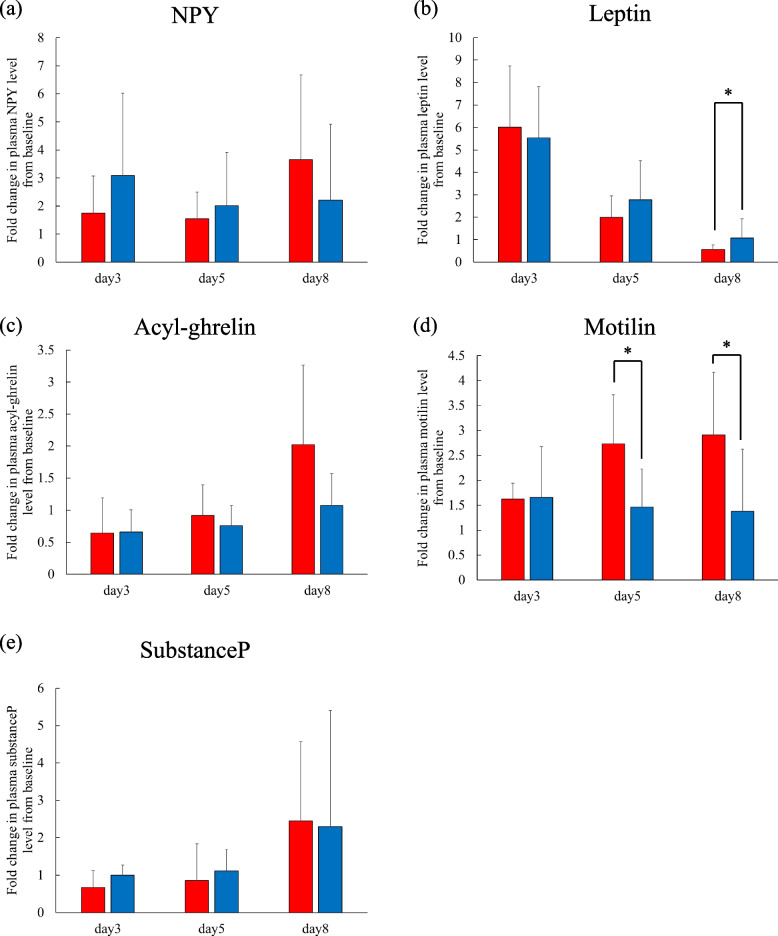
Comparisons of fold changes in plasma (**a**) NPY, (**b**) leptin, (**c**) acyl-ghrelin, (**d**) motilin, and (**e**) substance P levels with respect to baseline (day 1, before administration of chemotherapy) between the CINV (red columns; *n* = 4) and non-CINV (blue columns; *n* = 15) groups. Data are expressed as mean (column) ± standard deviation (vertical bar). Significant differences between groups were analyzed by Kruskal–Wallis test with Bonferroni correction. *p < 0.05, CINV vs. non-CINV. NPY, neuropeptide Y, CINV, chemotherapy-induced nausea and vomiting

Figure 3 shows the fold changes of plasma peptide levels in the CINV and non-CINV groups. Figure 4 compares the fold changes on days 3, 5 and 8 between the CINV and non-CINV groups. Plasma NPY increased significantly and acyl-ghrelin decreased significantly on day 3 compared to baseline in the non-CINV group, but these levels did not change significantly on day 3 compared to baseline in the CINV group (Fig. 3a and c). Comparisons of plasma NPY and acyl-ghrelin levels between the CINV and non-CINV groups showed no significant differences (Fig. 4a and c). Plasma leptin on day 8 was significantly higher in the non-CINV group than in the CINV group (Fig. 4b). Intriguingly, plasma motilin was significantly elevated on days 5 and 8 compared to baseline in the CINV group, but no such increases were observed in the non-CINV group (Fig. 3d), and plasma motilin levels were significantly higher in the CINV group than in the non-CINV group on days 5 and 8 (Fig. 4d).

### Differences in plasma gastrointestinal peptide levels following chemotherapy between anorexia and non-anorexia

**Fig. 5:**
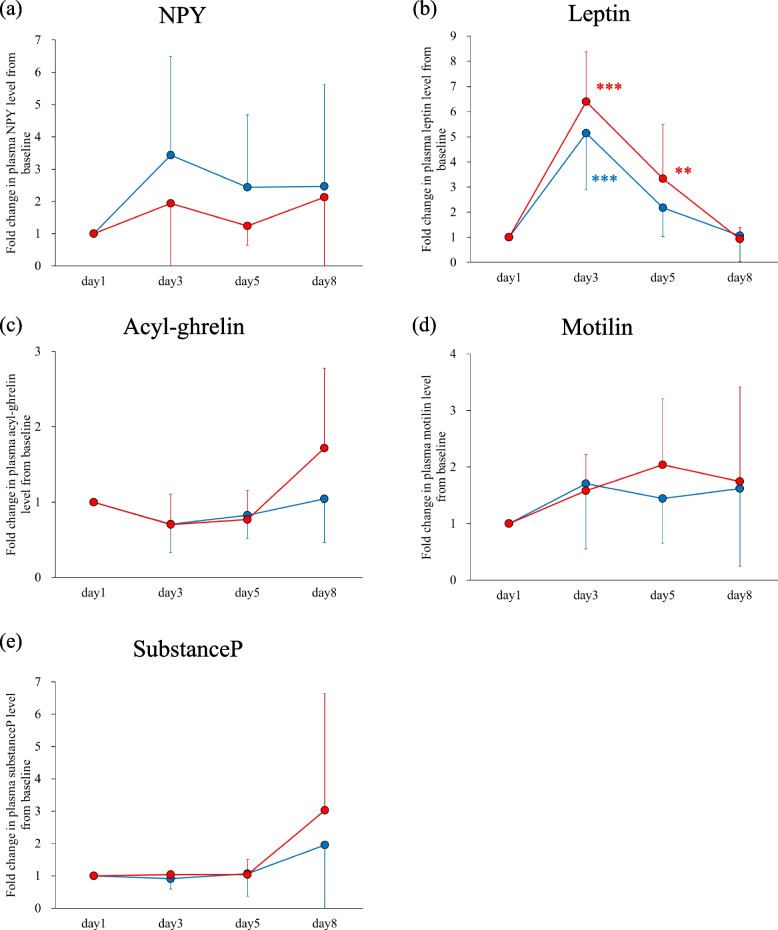
Fold changes in plasma (**a**) NPY, (**b**) leptin, (**c**) acyl-ghrelin, (**d**) motilin, and (**e**) substance P levels with respect to baseline (day 1, before administration of chemotherapy) in the anorexia (red circles and lines; *n* = 7) and non-anorexia (blue circles and lines; *n* = 10) groups. Data are expressed as mean (circle) ± standard deviation (vertical bar). Significant differences between groups were analyzed by Dunnett's test. **p < 0.01, ***p < 0.001, compared to day 1. NPY, neuropeptide Y

**Fig. 6:**
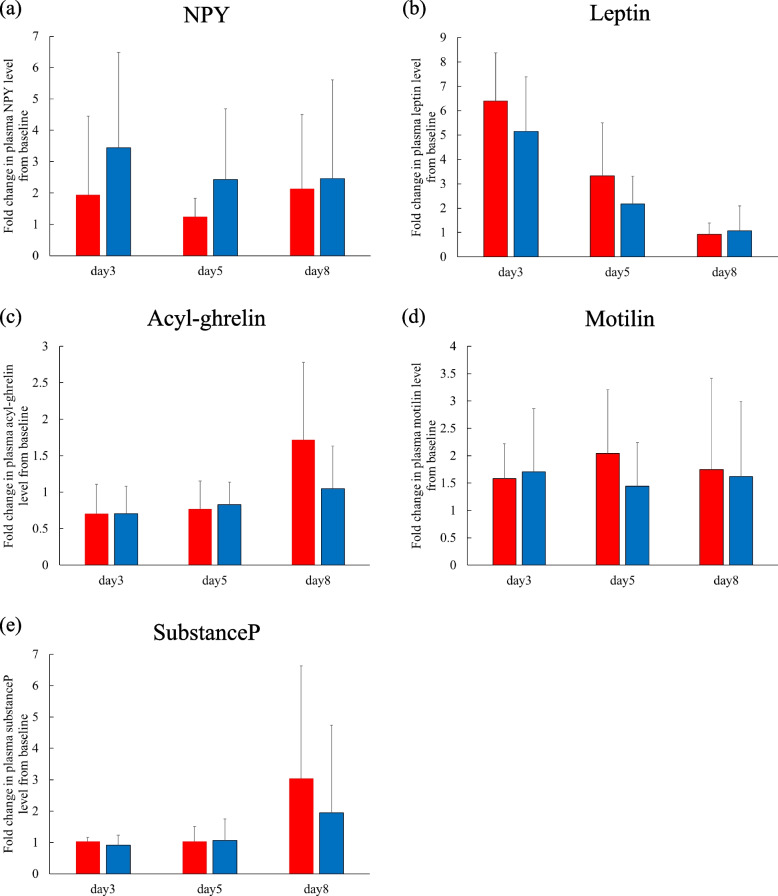
Comparisons of fold changed in plasma (**a**) NPY, (b) leptin, (**c**) acyl-ghrelin, (**d**) motilin, and (**e**) substance P levels with respect to baseline (day 1, before administration of chemotherapy) between the anorexia (red columns; *n* = 7) and non-anorexia (blue columns; *n* = 10) groups. Data are expressed as mean (column) ± standard deviation (vertical bar). Significant differences between groups were analyzed by Kruskal–Wallis test with Bonferroni correction. NPY, neuropeptide Y

Figure 5 shows the fold changes in plasma peptide levels in the anorexia and non-anorexia groups. Figure 6 compares the fold changes on days 3, 5 and 8 between the anorexia and non-anorexia groups. Plasma leptin was significantly elevated on day 3 compared to baseline in both groups; the level remained significantly higher on day 5 compared to baseline in the anorexia group, but was not significantly different from baseline in the non-anorexia group (Fig. 5b). No significant differences in all peptides were observed between the two groups at any point (Fig. 6).

## Discussion

In recent years, imbalance between gastrointestinal peptides such as acyl-ghrelin, motilin and NPY, and anorectic peptides such as pro-opiomelanocortin has been implicated as a cause of CINV and anorexia in cancer patients [11]. To date, only a few reports have examined the relationship between gastrointestinal peptides and CINV or post-chemotherapy anorexia, focusing on a single peptide or a few peptides [20, 21]. The present study comprehensively evaluated the changes in blood levels of plasma gastrointestinal peptides and the association with CINV and anorexia. The new findings are as follows: (1) plasma NPY and leptin were elevated and plasma acyl-ghrelin tended to decrease in the early phase of the chemotherapy session; (2) plasma motilin increased significantly on days 5 and 8 compared to baseline only in the CINV group, and the levels were significantly higher in the CINV group than in the non-CINV group; (3) plasma leptin remained significantly higher on day 5 compared to baseline only in the anorexia group.

Platinum-based drugs such as cisplatin cause many adverse events via 5-HT, such as renal damage, bone marrow suppression and digestive dysfunction. Cisplatin dose-dependently reduces plasma ghrelin level in rats, and the response is mediated by 5-HT3 or 5-HT4 receptors [22]. In our study, all patients received a 5-HT3 receptor antagonist (palonosetron) only for the first 24 h. Ten patients did not fulfill the definition of anorexia, but reduced appetite and decreased plasma ghrelin levels were observed from day 3 of the chemotherapy session, suggesting that the 5-HT3 receptor antagonist may be ineffective in preventing anorexia. Substance P and NK1 receptors are associated with late-onset CINV. Aprepitant inhibits the binding of substance P to the NK1 receptor in the vomiting center, and is effective for late-onset CINV that cannot be prevented by 5-HT3 receptor antagonists and manifests within 120 h after cancer chemotherapy [3, 4]. In this study, all patients received fosaprepitant prior to chemotherapy, but plasma substance P levels on days 3 and 5 did not increase compared to baseline. In addition, no significant differences in plasma substance P levels were observed between the CINV and non-CINV groups throughout the observation period. These findings suggest that CINV occurring in this study may be due to mechanisms other than the substance P–NK receptor pathway.

Plasma motilin levels increased significantly compared to baseline in the relatively late phase of the chemotherapy session only in the CINV group, and the levels were significantly higher in the CINV group than in the non-CINV group. Motilin is a hormone secreted by the upper gastrointestinal tract during fasting and is responsible for controlling the interdigestive MMC [23]. This hormone stimulates gastrointestinal peristalsis and promotes digestion, but excessive secretion enhances the interdigestive MMC causing nausea [24]. Therefore, the cause of CINV is speculated to be excessive secretion of motilin due to chemotherapy-induced loss of appetite.

Plasma acyl-ghrelin levels decreased following chemotherapy in 15 of 18 patients. Ghrelin has a variety of physiological activities and therefore has attracted attention in various fields. In particular, the effect of ghrelin in increasing food intake is well known, and the mechanism of action has been elucidated. Ghrelin is secreted by the stomach during fasting and stimulates food intake. In addition, signals are transmitted from the periphery to the brain via humoral or nervous pathways to suppress energy expenditure and maintain energy balance [25, 26]. Ghrelin affects the digestive system, stimulating gastric acid secretion and gastric emptying. Plasma acyl-ghrelin level increases before meal, peaks at the beginning of a meal, and returns to basal level within one hour after meal. Hiura et al. [20]. demonstrated that plasma total ghrelin levels decreased significantly on days 3 and 8 of cisplatin-based chemotherapy and were associated with cisplatin-induced anorexia. Rikkunshi-to, a traditional herbal medicine, increases plasma ghrelin level in rats and is effective against cisplatin-induced anorexia [22]. In a previous preliminary experimental study, electro-acupuncture was effective for chemotherapy-induced anorexia by upregulating plasma ghrelin level [27]. These effects may be associated with increased secretion of appetite-related peptides including ghrelin and NPY. However, in this study, no significant difference in plasma acyl-ghrelin level was observed between the anorexia and non-anorexia groups throughout the observation period.

Plasma level of leptin, an appetite-suppressing hormone, was significantly elevated on day 3, regardless of anorexia. However, the level remained significantly higher on day 5 compared to baseline in the anorexia group, whereas the level decreased and was not significantly different from baseline in the non-anorexia group. This finding suggests that anorexia may be related to the prolonged increase in leptin secretion in the late phase of the chemotherapy session. Leptin and ghrelin have opposite physiological functions in energy homeostasis: leptin inhibits food intake while ghrelin stimulates appetite [28]. Consistent with these characteristics, leptin and ghrelin levels fluctuated in opposite directions during the chemotherapy session. However, leptin does not regulate ghrelin secretion, and both peptides function independently in controlling energy homeostasis [28]. This may partially explain why, in contrast to leptin, no significant differences in acyl-ghrelin level were observed between the anorexia and non-anorexia groups.

The results of this study indicate that regulation of the substance P‒NK1 receptor pathway alone is not sufficient to suppress late-onset CINV. The present results also suggest that excessive motilin secretion may be responsible for the occurrence of nausea. Motilin is a peptide hormone that is secreted during fasting, and if anorexia occurs after chemotherapy, motilin secretion would be excessive and cause nausea. Two of the four patients who actually developed CINV had anorexia, and the other two had anorexia from baseline. Therefore, it is important to improve anorexia in order to control nausea, which may be achieved by using dopamine D2 receptor blockers such as metoclopramide and domperidone, or Rikkunshi-To.

Differences in patient-specific factors such as baseline nutritional status may also influence peptide secretion dynamics and the development of anorexia. The present data show a tendency of lower serum albumin levels in the anorexia group compared to the non-anorexia group (3.4 g/dL vs. 3.9 g/dL, *p* = 0.06). Previous reports have shown a negative correlation between albumin and leptin levels in the elderly and patients on dialysis [29, 30]. Taken together, these findings suggest that baseline nutritional status may contribute to the prolonged increase in leptin concentration observed in the anorexia group. In addition, although there are no reports of a correlation between albumin and motilin concentrations, when anorexia is accompanied by hunger, motilin is overproduced, which also leads to nausea. On the other hand, albumin level correlates inversely with leptin level, suggesting increased leptin secretion in a hypoalbuminemic state, causing anorexia. Thus, patients with low albumin levels may be more susceptible to develop anorexia and nausea.

This study had several limitations. First, in this single-center prospective cohort study in patients with esophageal cancer and urothelial cancer or testiculoma treated with a cisplatin-based regimen, only 20 patients were recruited during the study period. The small sample size may have resulted in insufficient statistical power to detect significant differences in plasma levels of peptides other than motilin and leptin between CINV and non-CINV groups, and between anorexia and non-anorexia groups. The small sample size was a key limitation of this study, which may have reduced the credibility of the results. While the detected effect sizes for the statistical tests were relatively high (Table S1), a large-scale multicenter study is needed to verify the present findings in the future. Second, we employed the VAS scale to score the severity of nausea and anorexia. The VAS scale was originally developed by Keel [31] as a simple descriptive pain scale. The VAS questionnaire has been modified to assess appetite, and the reproducibility and validity have been demonstrated [32, 33]. However, since this method depends on the subjective rating of the patient, it is undeniable that the score may be affected by the patients’ background when the number of samples is small. To address this problem, a large-scale study would also be necessary in the future. Third, the timing of cisplatin administration differs among chemotherapy regimens (CDDP/5-FU, day 1; CDDP + GEM, day 2; CDDP + BLM + ETP, days 1–5), which may influence the dynamics of gastrointestinal peptides, as well as the development of CINV and anorexia. However, a subanalysis of the effect of different chemotherapy regimens on the changes in plasma levels of the five peptides indicated minimal impact of CDDP schedule on the changes in peptide levels, with the exception of NPY (Figures S1-S5). Although CDDP + BLM + ETP includes five days of CDDP infusion, motilin levels remained almost stable during chemotherapy session, resulting in no patients developing CINV. Furthermore, although CDDP given on day 2 of GEM + CDDP therapy may conceivably shift the peak of motilin levels, no marked difference was observed visually between CDDP/5-FU and GEM + CDDP. Given the small number of patients who received GEM + CDDP or CDDP + BLM + ETP therapy and developed CINV or anorexia, further study is warranted to evaluate the impact of different chemotherapy regimens. Fourth, the number of chemotherapy cycles differed between patients. This variation may influence the development of CINV or anorexia, as these adverse events are more likely to occur during the first treatment cycle. However, no differences in changes in peptide levels between the first chemotherapy cycle and subsequent cycles were observed by visual assessment (Figure S6), suggesting minimal impact of chemotherapy cycle on the dynamics of gastrointestinal peptides. Since the number of study patients was limited, further study is warranted to evaluate the influence of chemotherapy cycle.

## Conclusions

This study investigated the relationship between gastrointestinal symptoms (CINV and anorexia) following chemotherapy and five types of gastrointestinal peptides, and demonstrated the association of CINV with excessive secretion of motilin, and that of anorexia with sustained elevation of leptin level. These results suggest the potential of motilin and leptin as quantitative indicators of CINV and anorexia.

## Supplementary Information

Supplementary Material 1.

## Data Availability

No datasets were generated or analysed during the current study.

## Abbreviations

CINV: Chemotherapy-induced nausea and vomiting
NK1: Neurokinin 1
VAS: Visual analog scale
5-HT3: 5-Hydroxytryptamine 3
CTCAE: Common Terminology Criteria for Adverse Events
MMC: Migrating motor complex
NPY: Neuropeptide Y
PS: Performance status
EDTA: Ethylenediaminetetraacetic acid
CDDP: Cisplatin

## References

[1] Hesketh PJ, Van Belle S, Aapro M, Tattersall FD, Naylor RJ, Hargreaves R, et al. Differential involvement of neurotransmitters through the time course of cisplatin-induced emesis as revealed by therapy with specific receptor antagonists. Eur J Cancer. 2003;39:1074–80. 12736106 10.1016/s0959-8049(02)00674-3

[2] Cubeddu LX, Hoffmann IS. Participation of serotonin on early and delayed emesis induced by initial and subsequent cycles of cisplatinum-based chemotherapy: effects of antiemetics. J Clin Pharmacol. 1993;33:691–7. 7691898 10.1002/j.1552-4604.1993.tb05608.x

[3] Hesketh PJ, Grunberg SM, Gralla RJ, Warr DG, Roila F, de Wit R, et al. The oral neurokinin-1 antagonist aprepitant for the prevention of chemotherapy-induced nausea and vomiting: a multinational, randomized, double-blind, placebo-controlled trial in patients receiving high-dose cisplatin–the aprepitant protocol 052 study group. J Clin Oncol. 2003;21:4112–9. 14559886 10.1200/JCO.2003.01.095

[4] Poli-Bigelli S, Rodrigues-Pereira J, Carides AD, Julie Ma G, Eldridge K, Hipple A, et al. Addition of the neurokinin 1 receptor antagonist aprepitant to standard antiemetic therapy improves control of chemotherapy-induced nausea and vomiting. Results from a randomized, double-blind, placebo-controlled trial in Latin America. Cancer. 2003;97:3090–8. 12784346 10.1002/cncr.11433

[5] Lindley CM, Hirsch JD, O’Neill CV, Transau MC, Gilbert CS, Osterhaus JT. Quality of life consequences of chemotherapy-induced emesis. Qual Life Res. 1992;1:331–40. 1299465 10.1007/BF00434947

[6] Kojima M, Hosoda H, Date Y, Nakazato M, Matsuo H, Kangawa K. Ghrelin is a growth-hormone-releasing acylated peptide from stomach. Nature. 1999;402:656–60. 10604470 10.1038/45230

[7] Müller TD, Perez-Tilve D, Tong J, Pfluger PT, Tschöp MH. Ghrelin and its potential in the treatment of eating/wasting disorders and cachexia. J Cachexia Sarcopenia Muscle. 2010;1:159–67. 21475701 10.1007/s13539-010-0012-4 PMC3060653

[8] Triantafyllou GA, Paschou SA, Mantzoros CS. Leptin and hormones: energy homeostasis. Endocrinol Metab Clin North Am. 2016;45:633–45. 27519135 10.1016/j.ecl.2016.04.012

[9] Koon HW, Pothoulakis C. Immunomodulatory properties of substance P: the gastrointestinal system as a model. Ann N Y Acad Sci. 2006;1088:23–40. 17192554 10.1196/annals.1366.024

[10] Grunberg SM, Deuson RR, Mavros P, Geling O, Hansen M, Cruciani G, et al. Incidence of chemotherapy-induced nausea and emesis after modern antiemetics. Cancer. 2004;100:2261–8. 15139073 10.1002/cncr.20230

[11] Sanger GJ, Broad J, Andrews PL. The relationship between gastric motility and nausea: gastric prokinetic agents as treatments. Eur J Pharmacol. 2013;715:10–4. 23831391 10.1016/j.ejphar.2013.06.031

[12] Brown JC, Cook MA, Dryburgh JR. Motilin, a gastric motor activity-stimulating polypeptide: final purification, amino acid composition, and C-terminal residues. Gastroenterology. 1972;62:401–4. 5011531

[13] Deloose E, Janssen P, Depoortere I, Tack J. The migrating motor complex: control mechanisms and its role in health and disease. Nat Rev Gastroenterol Hepatol. 2012;9:271–85. 22450306 10.1038/nrgastro.2012.57

[14] Wilding JP. Neuropeptides and appetite control. Diabet Med. 2002;19:619–27. 12147141 10.1046/j.1464-5491.2002.00790.x

[15] Tatemoto K, Carlquist M, Mutt V. Neuropeptide Y–a novel brain peptide with structural similarities to peptide YY and pancreatic polypeptide. Nature. 1982;296:659–60. 6896083 10.1038/296659a0

[16] Allen YS, Adrian TE, Allen JM, Tatemoto K, Crow TJ, Bloom SR, et al. Neuropeptide Y distribution in the rat brain. Science. 1983;221:877–9. 6136091 10.1126/science.6136091

[17] Aogi K, Takeuchi H, Saeki T, Aiba K, Tamura K, Iino K, et al. Optimizing antiemetic treatment for chemotherapy-induced nausea and vomiting in Japan: update summary of the 2015 Japan Society of Clinical Oncology clinical practice guidelines for antiemesis. Int J Clin Oncol. 2021;26:1–17. 33161452 10.1007/s10147-020-01818-3 PMC7788035

[18] Baba Y, Baba H, Yamamoto S, Shimada H, Shibata T, Miyazaki T, et al. Chemotherapy-induced nausea and vomiting is less controlled at delayed phase in patients with esophageal cancer: a prospective registration study by the CINV study group of Japan. Dis Esophagus. 2017;30:1–7. 10.1111/dote.12482 27001532

[19] Mizuno M, Hiura M, Kikkawa F, Numa F, Yaegashi N, Narahara H, et al. A prospective observational study on chemotherapy-induced nausea and vomiting (CINV) in patients with gynecologic cancer by the CINV study group of Japan. Gynecol Oncol. 2016;140:559–64. 26748216 10.1016/j.ygyno.2015.12.029

[20] Hiura Y, Takiguchi S, Yamamoto K, Kurokawa Y, Yamasaki M, Nakajima K, et al. Fall in plasma ghrelin concentrations after cisplatin-based chemotherapy in esophageal cancer patients. Int J Clin Oncol. 2012;17:316–23. 21773688 10.1007/s10147-011-0289-0

[21] Takahashi T, Nakamura Y, Tsuya A, Murakami H, Endo M, Yamamoto N. Pharmacokinetics of aprepitant and dexamethasone after administration of chemotherapeutic agents and effects of plasma substance P concentration on chemotherapy-induced nausea and vomiting in Japanese cancer patients. Cancer Chemother Pharmacol. 2011;68:653–9. 21125277 10.1007/s00280-010-1519-2 PMC3162145

[22] Takeda H, Sadakane C, Hattori T, Katsurada T, Ohkawara T, Nagai K, et al. Rikkunshito, an herbal medicine, suppresses cisplatin-induced anorexia in rats via 5-HT2 receptor antagonism. Gastroenterology. 2008;134:2004–13. 18439428 10.1053/j.gastro.2008.02.078

[23] Janssens J, Vantrappen G, Peeters TL. The activity front of the migrating motor complex of the human stomach but not of the small intestine is motilin-dependent. Regul Pept. 1983;6:363–9. 6635258 10.1016/0167-0115(83)90265-3

[24] Sarna SK, Soergel KH, Koch TR, Stone JE, Wood CM, Ryan RP, et al. Gastrointestinal motor effects of erythromycin in humans. Gastroenterology. 1991;101:1488–96. 1955115 10.1016/0016-5085(91)90383-v

[25] Nakazato M, Murakami N, Date Y, Kojima M, Matsuo H, Kangawa K, et al. A role for ghrelin in the central regulation of feeding. Nature. 2001;409:194–8. 11196643 10.1038/35051587

[26] van der Lely AJ, Tschöp M, Heiman ML, Ghigo E. Biological, physiological, pathophysiological, and pharmacological aspects of ghrelin. Endocr Rev. 2004;25:426–57. 15180951 10.1210/er.2002-0029

[27] Kang KS, Huh W, Bang Y, Choi HJ, Baek JY, Song JH, et al. Electroacupuncture for chemotherapy-induced anorexia through humoral appetite regulation: a preliminary experimental study. Exp Ther Med. 2019;17:2587–97. 30906450 10.3892/etm.2019.7250 PMC6425152

[28] Klok MD, Jakobsdottir S, Drent ML. The role of leptin and ghrelin in the regulation of food intake and body weight in humans: a review. Obes Rev. 2007;8:21–34. 17212793 10.1111/j.1467-789X.2006.00270.x

[29] Bouillanne O, Golmard JL, Coussieu C, Noël M, Durand D, Piette F, et al. Leptin a new biological marker for evaluating malnutrition in elderly patients. Eur J Clin Nutr. 2007;61:647–54. 17151588 10.1038/sj.ejcn.1602572

[30] Johansen KL, Mulligan K, Tai V, Schambelan M. Leptin, body composition, and indices of malnutrition in patients on dialysis. J Am Soc Nephrol. 1998;9:1080–4. 9621292 10.1681/ASN.V961080

[31] Keele KD. Pain-sensitivity tests; the pressure algometer. Lancet. 1954;266:636–9. 13143740 10.1016/s0140-6736(54)92347-8

[32] Parker BA, Sturm K, MacIntosh CG, Feinle C, Horowitz M, Chapman IM. Relation between food intake and visual analogue scale ratings of appetite and other sensations in healthy older and young subjects. Eur J Clin Nutr. 2004;58:212–8. 14749739 10.1038/sj.ejcn.1601768

[33] Flint A, Raben A, Blundell JE, Astrup A. Reproducibility, power and validity of visual analogue scales in assessment of appetite sensations in single test meal studies. Int J Obes Relat Metab Disord. 2000;24:38–48. 10702749 10.1038/sj.ijo.0801083

