# Supplementary material for: Association of chemotherapy-induced nausea and vomiting or anorexia with plasma levels of five gastrointestinal peptides in patients receiving chemotherapy
Source: J Pharm Health Care Sci. 2025 Mar 5;11:17. doi: 10.1186/s40780-025-00424-7 (PMC11881272; doi:10.1186/s40780-025-00424-7)
Supplement: Supplementary file 1 — Supplementary Material 1. [file 40780_2025_424_MOESM1_ESM.docx]

**Supplemental Data**

**Supplemental Table S1.** The p-values and effect sizes of all the statistical tests. The effect sizes were calculated using Cohen’s d. CINV, chemotherapy-induced nausea and vomiting.

| Figure | Analysis | *p*-value | Effect size | 95% confidence interval |
| --- | --- | --- | --- | --- |
| 2a | Day 1 vs 3 | 0.028 | 0.942 | 0.287 to 1.597 |
|  | Day 1 vs 5 | 0.386 | 0.767 | 0.124 to 1.411 |
|  | Day 1 vs 8 | 0.073 | 0.789 | 0.144 to 1.434 |
| 2b | Day 1 vs 3 | <0.001 | 3.009 | 2.092 to 3.927 |
|  | Day 1 vs 5 | 0.002 | 1.441 | 0.741 to 2.140 |
|  | Day 1 vs 8 | 0.999 | 0.092 | -0.528 to 0.712 |
| 2c | Day 1 vs 3 | 0.079 | 1.339 | 0.612 to 2.066 |
|  | Day 1 vs 5 | 0.381 | 0.917 | 0.228 to 1.606 |
|  | Day 1 vs 8 | 0.214 | 0.496 | -0.168 to 1.160 |
| 2d | Day 1 vs 3 | 0.068 | 1.088 | 0.422 to 1.755 |
|  | Day 1 vs 5 | 0.043 | 1.136 | 0.465 to 1.806 |
|  | Day 1 vs 8 | 0.058 | 0.739 | 0.098 to 1.381 |
| 2e | Day 1 vs 3 | 0.998 | 0.303 | -0.354 to 0.961 |
|  | Day 1 vs 5 | 1.000 | 0.154 | -0.500 to 0.809 |
|  | Day 1 vs 8 | 0.036 | 0.658 | -0.023 to 1.340 |
| 3a | CINV Day 1 vs 3 | 0.869 | 0.927 | -0.555 to 2.408 |
|  | CINV Day 1 vs 5 | 0.940 | 0.960 | -0.529 to 2.449 |
|  | CINV Day 1 vs 8 | 0.117 | 1.438 | -0.169 to 3.045 |
|  | non-CINV Day 1 vs 3 | 0.033 | 1.036 | 0.271 to 1.802 |
|  | non-CINV Day 1 vs 5 | 0.435 | 0.755 | 0.012 to 1.497 |
|  | non-CINV Day 1 vs 8 | 0.299 | 0.631 | -0.103 to 1.366 |
| 3b | CINV Day 1 vs 3 | 0.001 | 3.007 | 0.813 to 5.202 |
|  | CINV Day 1 vs 5 | 0.655 | 1.688 | 0.005 to 3.372 |
|  | CINV Day 1 vs 8 | 0.947 | 3.434 | 1.047 to 5.820 |
|  | non-CINV Day 1 vs 3 | <0.001 | 2.940 | 1.889 to 3.991 |
|  | non-CINV Day 1 vs 5 | 0.004 | 1.492 | 0.676 to 2.307 |
|  | non-CINV Day 1 vs 8 | 0.999 | 0.112 | -0.605 to 0.828 |
| 3c | CINV Day 1 vs 3 | 0.826 | 1.057 | -0.453 to 2.566 |
|  | CINV Day 1 vs 5 | 0.997 | 0.273 | -1.122 to 1.667 |
|  | CINV Day 1 vs 8 | 0.160 | 1.341 | -0.239 to 2.921 |
|  | non-CINV Day 1 vs 3 | 0.016 | 1.436 | 0.627 to 2.244 |
|  | non-CINV Day 1 vs 5 | 0.096 | 1.324 | 0.529 to 2.120 |
|  | non-CINV Day 1 vs 8 | 0.945 | 0.217 | -0.501 to 0.935 |
| 3d | CINV Day 1 vs 3 | 0.582 | 3.283 | 0.965 to 5.600 |
|  | CINV Day 1 vs 5 | 0.028 | 2.880 | 0.741 to 5.019 |
|  | CINV Day 1 vs 8 | 0.016 | 2.472 | 0.503 to 4.441 |
|  | non-CINV Day 1 vs 3 | 0.079 | 0.945 | 0.188 to 1.702 |
|  | non-CINV Day 1 vs 5 | 0.299 | 0.878 | 0.126 to 1.630 |
|  | non-CINV Day 1 vs 8 | 0.467 | 0.432 | -0.293 to 1.157 |
| 3e | CINV Day 1 vs 3 | 0.966 | 1.252 | -0.305 to 2.808 |
|  | CINV Day 1 vs 5 | 0.997 | 0.252 | -1.141 to 1.646 |
|  | CINV Day 1 vs 8 | 0.285 | 1.181 | -0.357 to 2.720 |
|  | non-CINV Day 1 vs 3 | 1.000 | 0.000 | -0.715 to 0.716 |
|  | non-CINV Day 1 vs 5 | 0.994 | 0.276 | -0.443 to 0.996 |
|  | non-CINV Day 1 vs 8 | 0.077 | 0.909 | 0.154 to 1.663 |
| 4a | Day 3 CINV vs non-CINV | 0.764 | 0.507 | -0.609 to 1.623 |
|  | Day 5 CINV vs non-CINV | 0.920 | 0.262 | -0.845 to 1.368 |
|  | Day 8 CINV vs non-CINV | 0.271 | 0.540 | -0.578 to 1.657 |
| 4b | Day 3 CINV vs non-CINV | 0.764 | 0.209 | -0.896 to 1.315 |
|  | Day 5 CINV vs non-CINV | 0.317 | 0.497 | -0.618 to 1.613 |
|  | Day 8 CINV vs non-CINV | 0.046 | 0.656 | -0.469 to 1.781 |
| 4c | Day 3 CINV vs non-CINV | 0.821 | 0.039 | -1.064 to 1.142 |
|  | Day 5 CINV vs non-CINV | 0.258 | 0.557 | -0.561 to 1.676 |
|  | Day 8 CINV vs non-CINV | 0.141 | 1.497 | 0.285 to 2.709 |
| 4d | Day 3 CINV vs non-CINV | 0.484 | 0.036 | -1.067 to 1.139 |
|  | Day 5 CINV vs non-CINV | 0.028 | 1.638 | 0.405 to 2.870 |
|  | Day 8 CINV vs non-CINV | 0.036 | 1.270 | 0.088 to 2.453 |
| 4e | Day 3 CINV vs non-CINV | 0.130 | 1.164 | -0.007 to 2.334 |
|  | Day 5 CINV vs non-CINV | 0.529 | 0.411 | -0.700 to 1.523 |
|  | Day 8 CINV vs non-CINV | 0.737 | 0.075 | -1.028 to 1.178 |
| 5a | Anorexia Day 1 vs 3 | 0.515 | 0.574 | -0.499 to 1.646 |
|  | Anorexia Day 1 vs 5 | 0.902 | 0.620 | -0.457 to 1.696 |
|  | Anorexia Day 1 vs 8 | 0.344 | 0.732 | -0.356 to 1.82 |
|  | non-anorexia Day 1 vs 3 | 0.057 | 1.193 | 0.233 to 2.152 |
|  | non-anorexia Day 1 vs 5 | 0.450 | 0.956 | 0.025 to 1.886 |
|  | non-anorexia Day 1 vs 8 | 0.207 | 0.696 | -0.210 to 1.601 |
| 5b | Anorexia Day 1 vs 3 | <0.001 | 4.162 | 2.195 to 6.129 |
|  | Anorexia Day 1 vs 5 | 0.007 | 1.643 | 0.406 to 2.88 |
|  | Anorexia Day 1 vs 8 | 0.997 | 0.247 | -0.805 to 1.299 |
|  | non-anorexia Day 1 vs 3 | <0.001 | 2.759 | 1.502 to 4.016 |
|  | non-anorexia Day 1 vs 5 | 0.158 | 1.530 | 0.521 to 2.539 |
|  | non-anorexia Day 1 vs 8 | 1.000 | 0.094 | -0.783 to 0.971 |
| 5c | Anorexia Day 1 vs 3 | 0.464 | 1.138 | -0.004 to 2.281 |
|  | Anorexia Day 1 vs 5 | 0.783 | 0.927 | -0.185 to 2.038 |
|  | Anorexia Day 1 vs 8 | 0.083 | 1.046 | -0.082 to 2.174 |
|  | non-anorexia Day 1 vs 3 | 0.095 | 1.167 | 0.211 to 2.123 |
|  | non-anorexia Day 1 vs 5 | 0.400 | 0.829 | -0.089 to 1.746 |
|  | non-anorexia Day 1 vs 8 | 0.999 | 0.119 | -0.759 to 0.996 |
| 5d | Anorexia Day 1 vs 3 | 0.321 | 1.380 | 0.196 to 2.564 |
|  | Anorexia Day 1 vs 5 | 0.106 | 1.366 | 0.184 to 2.547 |
|  | Anorexia Day 1 vs 8 | 0.269 | 0.681 | -0.401 to 1.764 |
|  | non-anorexia Day 1 vs 3 | 0.253 | 0.911 | -0.014 to 1.837 |
|  | non-anorexia Day 1 vs 5 | 0.406 | 0.840 | -0.078 to 1.759 |
|  | non-anorexia Day 1 vs 8 | 0.258 | 0.671 | -0.232 to 1.575 |
| 5e | Anorexia Day 1 vs 3 | 1.000 | 0.437 | -0.625 to 1.500 |
|  | Anorexia Day 1 vs 5 | 0.995 | 0.120 | -0.928 to 1.169 |
|  | Anorexia Day 1 vs 8 | 0.050 | 0.874 | -0.231 to 1.978 |
|  | non-anorexia Day 1 vs 3 | 0.992 | 0.407 | -0.480 to 1.293 |
|  | non-anorexia Day 1 vs 5 | 1.000 | 0.143 | -0.735 to 1.021 |
|  | non-anorexia Day 1 vs 8 | 0.504 | 0.557 | -0.338 to 1.453 |
| 6a | Day 3 anorexia vs non-anorexia | 0.051 | 0.559 | -0.427 to 1.546 |
|  | Day 5 anorexia vs non-anorexia | 0.143 | 0.711 | -0.288 to 1.709 |
|  | Day 8 anorexia vs non-anorexia | 0.495 | 0.124 | -0.843 to 1.091 |
| 6b | Day 3 anorexia vs non-anorexia | 0.283 | 0.620 | -0.371 to 1.611 |
|  | Day 5 anorexia vs non-anorexia | 0.329 | 0.761 | -0.243 to 1.765 |
|  | Day 8 anorexia vs non-anorexia | 1.000 | 0.174 | -0.794 to 1.142 |
| 6c | Day 3 anorexia vs non-anorexia | 0.906 | 0.004 | -0.962 to 0.970 |
|  | Day 5 anorexia vs non-anorexia | 0.637 | 0.183 | -0.785 to 1.152 |
|  | Day 8 anorexia vs non-anorexia | 0.077 | 0.897 | -0.120 to 1.915 |
| 6d | Day 3 anorexia vs non-anorexia | 0.696 | 0.136 | -0.831 to 1.103 |
|  | Day 5 anorexia vs non-anorexia | 0.143 | 0.665 | -0.330 to 1.660 |
|  | Day 8 anorexia vs non-anorexia | 0.770 | 0.089 | -0.877 to 1.056 |
| 6e | Day 3 anorexia vs non-anorexia | 0.768 | 0.500 | -0.482 to 1.482 |
|  | Day 5 anorexia vs non-anorexia | 1.000 | 0.047 | -0.919 to 1.013 |
|  | Day 8 anorexia vs non-anorexia | 0.302 | 0.385 | -0.591 to 1.360 |

**Supplemental Figure S1.** Fold changes in plasma neuropeptide Y level with respect to baseline in (a) all patients (n = 20) and in patients receiving (b) CDDP/5-FU (n = 10), (c) CDDP+GEM (n = 8), and (d) CDDP+BLM+ETP (n = 2). Dotted lines represent changes in individual patients. Group data are expressed as mean (yellow circle) ± standard deviation (vertical bar). CDDP, cisplatin; 5-FU, 5-fluorouracil; GEM, gemcitabine; BLM, bleomycin; ETP, etoposide.

**Supplemental Figure S2.** Fold changes in plasma leptin level with respect to baseline in (a) all patients (n = 20) and in patients receiving (b) CDDP/5-FU (n = 10), (c) CDDP+GEM (n = 8), and (d) CDDP+BLM+ETP (n = 2). Dotted lines represent changes in individual patients. Group data are expressed as mean (yellow circle) ± standard deviation (vertical bar). CDDP, cisplatin; 5-FU, 5-fluorouracil; GEM, gemcitabine; BLM, bleomycin; ETP, etoposide.

**Supplemental Figure S3.** Fold changes in plasma acyl-ghrelin level with respect to baseline in (a) all patients (n = 20) and in patients receiving (b) CDDP/5-FU (n = 10), (c) CDDP+GEM (n = 8), and (d) CDDP+BLM+ETP (n = 2). Dotted lines represent changes in individual patients. Group data are expressed as mean (yellow circle) ± standard deviation (vertical bar). CDDP, cisplatin; 5-FU, 5-fluorouracil; GEM, gemcitabine; BLM, bleomycin; ETP, etoposide.

**Supplemental Figure S4.** Fold changes in plasma motilin level with respect to baseline in (a) all patients (n = 20) and in patients receiving (b) CDDP/5-FU (n = 10), (c) CDDP+GEM (n = 8), and (d) CDDP+BLM+ETP (n = 2). Dotted lines represent changes in individual patients. Group data are expressed as mean (yellow circle) ± standard deviation (vertical bar). CDDP, cisplatin; 5-FU, 5-fluorouracil; GEM, gemcitabine; BLM, bleomycin; ETP, etoposide.

**Supplemental Figure S5.** Fold changes in plasma substance P level with respect to baseline in (a) all patients (n = 20) and in patients receiving (b) CDDP/5-FU (n = 10), (c) CDDP+GEM (n = 8), and (d) CDDP+BLM+ETP (n = 2). Dotted lines represent changes in individual patients. Group data are expressed as mean (yellow circle) ± standard deviation (vertical bar). CDDP, cisplatin; 5-FU, 5-fluorouracil; GEM, gemcitabine; BLM, bleomycin; ETP, etoposide.

**Supplemental Figure 6.** Fold changes in plasma (a, b) NPY, (c, d) leptin, (e, f) acyl-ghrelin, (g, h) motilin, and (i, j) substance P levels with respect to baseline in patients receiving (a, c, e, g, i) the first chemotherapy cycle (n = 8) and (b, d, f, h, j) subsequent cycle (n = 12). Dotted lines represent changes in individual patients. Group data are expressed as mean (yellow circle) ± standard deviation (vertical bar). NPY, neuropeptide Y.
